# Hematopoietically-Expressed Homeobox Gene Three Widely-Evaluated Polymorphisms and Risk for Diabetes: A Meta-Analysis

**DOI:** 10.1371/journal.pone.0049917

**Published:** 2012-11-15

**Authors:** Xiaobo Li, Yuqiong Li, Bei Song, Shujie Guo, Shaoli Chu, Nan Jia, Wenquan Niu

**Affiliations:** 1 State Key Laboratory of Medical Genomics, Ruijin Hospital, Shanghai Jiao Tong University School of Medicine, Shanghai, China; 2 Department of Hypertension, Ruijin Hospital, Shanghai Jiao Tong University School of Medicine, Shanghai, China; 3 Shanghai Institute of Hypertension, Shanghai, China; Thomas Jefferson University, United States of America

## Abstract

**Background:**

The hematopoietically-expressed homeobox (*HHEX*) gene is identified as a promising candidate for type 2 diabetes by genome-wide association studies, triggering plenty of subsequent replications; however, the results are conflicting. We therefore conducted a meta-analysis of three widely-evaluated polymorphisms in *HHEX* gene and diabetes risk.

**Methodology/Principal Findings:**

A random-effects model was adopted irrespective of heterogeneity. Data and study quality were assessed in duplicate. There were 49 studies (cases/controls: 57931/74658) for rs1111875, 18 studies (18227/30366) for rs5015480 and 26 studies (25725/30579) for rs7923837, respectively. Overall analyses indicated that rs1111875-C allele (odds ratio [OR] = 1.16; 95% confidence interval [CI]: 1.13–1.2; P<0.0005), rs5015480-C allele (OR = 1.16; 95% CI: 1.06–1.26; P = 0.001) and rs7923837-G allele (OR = 1.18; 95% CI: 1.12–1.24; P<0.0005) conferred significantly increased risk for type 2 diabetes, yet accompanying moderate to strong evidence of heterogeneity. Despite vast divergence in allele distributions, subgroup analyses by ethnicity showed comparable risk estimates between Asians and Caucasians for three examined polymorphisms. Moreover, results of studies with hospital-based controls deviated greatly from that of all qualified studies, especially for rs7923837-G allele carrying a doubled risk (OR = 1.37 versus 1.18). Furthermore, when only large studies (≥500 case-patients) were considered, risk effects were identical to the overall estimates for three examined polymorphisms. The Begg's funnel plot and Egger's test indicated low probability of publication bias.

**Conclusions:**

Our results provide clarification to the significant association of rs1111875, rs5015480 and rs7923837 in *HHEX* gene with type 2 diabetes.

## Introduction

The gene encoding hematopoietically-expressed homeobox (*HHEX*) has been repeatedly identified as a promising candidate in susceptibility to type 2 diabetes by genome-wide association studies across ethnicities [1]–[6]. As a member of homeobox family, *HHEX* gene encodes a transcription factor involved in a fundamental pathway (known as Wnt signaling) for cell growth and development [7]. *HHEX* gene is located on chromosome 10q24, contains 4 exons, and spans about 5.7 kb of genomic sequence [8]. Converging interest in unraveling the genetic determinants of *HHEX* attributable to the development of hyperglycemia has triggered hundreds of association studies lately. Several polymorphisms in *HHEX* gene have been identified, with rs1111875 T>C, rs5015480 T>C and rs7923837 A>G being extensively evaluated. However, results are controversial and firm association has not yet been established, possibly due to some methodological drawbacks, including the insufficient sample sizes, inappropriate selection of patients and controls, population stratification/admixture, ethnicity-specific genetic backgrounds, and lack of adjustment for confounders [9].

To shed some light on these controversial issues and to quantify risk estimates reliably, we conducted a meta-analysis of all available studies relating three aforementioned polymorphisms in *HHEX* gene to the risk of developing diabetes, and explored the potential sources of between-study heterogeneity, and the possible existence of publication bias.

## Methods

This meta-analysis is reported in accordance with the Preferred Reporting Items for Systematic Reviews and Meta-analyses (PRISMA) statement [10].

### Search strategy

We conducted a comprehensive search of PubMed database from its inception through March 2012 using the following subject terms: hematopoietically-expressed homeobox, *HHEX*, diabetes, gene and polymorphism. Articles written in English and performed on humans were identified. We browsed the title and abstract of all retrieved articles to determine whether data on the topic of interest were included. If article could not be rejected with certainty, we resorted to the full text of article for evaluation. The search was also supplemented by checking the bibliographies from the main reports and relevant reviews for additional eligible articles unidentified by PubMed. We abstracted the most complete data from articles with duplicate or overlapping samples. We treated articles that had more than one subgroup with homogenous characteristics separately.

### Inclusion/exclusion criteria

The quantified articles must meet the following criteria: (1) evaluating the relationship of either rs1111875 or rs5015480 or rs7923837 in *HHEX* gene with diabetes risk; (2) involving unrelated subjects and non-overlapping data; (3) having a retrospective or nested case-control design; (4) genotyping with validated methods; (5) supplying genotype/allele counts between cases and controls or the odds ratio (OR) and its 95% confidence interval (95% CI) of examined polymorphisms. Articles investigating phenotype modification, response to treatment, birth weight, insulin secretion, β-cell function, survival or family-based studies were excluded. Moreover, case reports/series, editorials, review articles, and non-English articles were also excluded.

### Data extraction

All data were reviewed and extracted independently by two investigators (X.L. and Y.L.) using a standardized data-extraction form. The following data were collected from each article: the first author's name, publication year, ethnicity of study population, types of diabetes and/or complications, diagnostic criteria, study design, source of controls, genotyping method, genotypes or alleles of examined polymorphisms, and other traditional risk factors, if available, including age, percentage of males and body mass index (BMI). Any discrepancies were resolved by discussion and, when necessary, adjudicated by a third investigator (W.N.).

### Statistical analysis

Due to a considerable number of articles providing data only on allele counts and to enhance study power to detect an association, we exclusively took account of allelic model in this meta-analysis. Pooled OR (95% CI) for diabetes risk associated with *HHEX* gene rs1111875-C, rs5015480-C and rs7923837-G alleles compared with the alternative alleles were calculated, respectively. The goodness-of-fit of the observed allele frequencies with the expected frequencies by Hardy-Weinberg equilibrium was assessed using the χ^2^ test or Fisher's exact test. Between-group differences of demographic data were calculated by the Student's paired t test.

We employed a random-effects model to bring the individual effect size estimates together, and quantified between-study heterogeneity by inconsistency index (*I* ^2^) statistic (ranging from 0 to 100%). The *I* ^2^ statistic is defined as the percentage of the observed between-study variability that is due to heterogeneity rather than chance, with high values suggesting more possible existence of heterogeneity. Potential heterogeneity between results of individual studies or in subgroups respectively by disease subtype, ethnicity, study design, source of controls, genotyping method, diagnostic criterion, and sample size was explored using χ^2^ test.

A cumulative analysis was performed according to the ascending year of publication to identity the evolution of the combined estimates over time. Complete data on age, gender and BMI were only available in a subset of articles, and to estimate potential confounders on the relationship between *HHEX* gene and diabetes risk, a multivariable meta-regression model was employed. Publication bias was assessed using the Egger regression test. The Egger's test detects Begg's funnel plot asymmetry by determining whether the intercept deviates significantly from zero in a regression of the standardized effect estimates against their precision.

Significance was judged at P<0.05, with exceptions of the *I* ^2^ statistic and Egger's test at P<0.1 [11]. Data management and statistical analyses were conducted using STATA software version 11.2 (StataCorp LP, College Station, TX, USA) for Windows.

## Results

### Characteristics of the included studies

**Figure 1:**
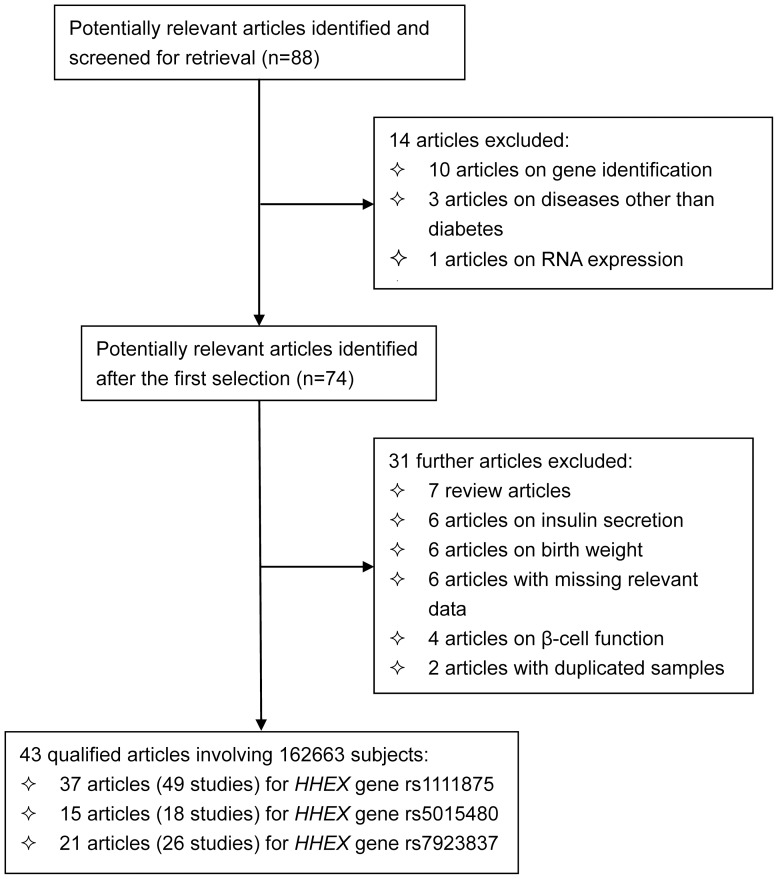
Flow diagram of search strategy and study selection.

The initial search generated 88 articles, and 43 quantified articles [2]–[4], [12]–[47] involving 162663 subjects met our selection criteria. A flow diagram schematizing the selection process of identified articles with specific reasons, and the baseline characteristics of all qualified studies are presented in Figure 1 and Table 1, respectively. In addition to type 2 diabetes, five articles provided data on impaired glucose tolerance (IGT) or impaired fasting glucose (IFG) [41], [42], [44], [45], as two forms of pre-diabetes, whose association was synthesized as well. Since 10 articles involved subjects of more than one homozygous group, a total of 56 studies were analyzed finally, with 34 studies in Asians, 18 in Caucasians, 2 in Africans, 1 in Arabs, and 1 in Mexicans. Forty-eight of 56 studies were retrospective in design, and 8 were prospective. Ten studies recruited controls from hospitals, and the rest 46 studies from general populations. As for genotyping methods, 26 studies adopted TaqMan technique, 23 studies Array technique, 4 studies PCR-based technique, 2 studies SNPshot, 1 study SNPlex technique. Out of 40 studies with complete diagnostic information on diabetes, 37 studies met up with the WHO criteria, and 3 studies with the ADA criteria. The mean levels of age (P = 0.013), male percentage (P<0.0005) and BMI (P<0.0005) were significant higher in cases than in controls.

**Table 1:** The baseline characteristics of all qualified studies in this meta-analysis.

Author & year	Ethnicity	Type	Design	Source	Genotyping	Diagnosis	Age, years		Gender (Males)		BMI, kg/m^2^	
							Cases	Controls	Cases	Controls	Cases	Controls
Grarup N (2007)	Caucasian	T2DM	Retros.	PBC	TaqMan	WHO	46.2	25.3	0.475	0.593	NA	NA
Horikoshi M (2007)	Asian	T2DM	Retros.	PBC	TaqMan	NA	63.1	69.5	0.619	0.447	24.3	23.8
Schulze MB (2007)	Caucasian	T2DM	Pros.	PBC	TaqMan	WHO	NA	NA	NA	NA	NA	NA
Scott L (2007)	Caucasian	T2DM	Retros.	PBC	ARRAY	WHO	63.4	64.0	0.58	0.552	29.8	26.8
Sladek R (2007)	Caucasian	T2DM	Retros.	PBC	ARRAY	ADA	60.8	56.4	0.617	0.068	28.9	25.3
Zeggini E (2007) a	Caucasian	T2DM	Pros.	PBC	ARRAY	NA	58.6	NA	0.581	0.492	30.7	NA
Zeggini E (2007) b	Caucasian	T2DM	Retros.	PBC	ARRAY	NA	64.2	58.7	0.579	0.512	31.0	26.3
Zeggini E (2007) c	Caucasian	T2DM	Retros.	PBC	ARRAY	NA	58.4	31.6	0.583	0.489	31.5	25.0
Groenewoud MJ (2008) a	Caucasian	IGT/IFG	Retros.	PBC	TaqMan	NA	57.0	45.8	0.479	0.239	28.4	25.8
Groenewoud MJ (2008) b	Caucasian	IGT/IFG	Retros.	PBC	TaqMan	NA	45.2	36.6	0.486	0.41	26.9	25.0
Furukawa Y (2008)	Asian	T2DM	Retros.	HBC	TaqMan	WHO	64.8	50.0	0.551	NA	23.9	22.3
Herder C (2008)	Caucasian	T2DM	Pros.	PBC	ARRAY	NA	65.2	61.9	0.589	0.482	30.9	27.7
Horikawa Y (2008)	Asian	T2DM	Retros.	HBC	TaqMan	NA	NA	NA	NA	NA	NA	NA
Lee Y (2008)	Asian	T2DM	Retros.	HBC	TaqMan	NA	58.2	55.0	0.516	0.464	24.3	22.1
Lewis J (2008)	African	T2DM	Retros.	PBC	ARRAY	NA	61.8	51.4	0.37	0.45	NA	NA
Ng MC (2008)	Asian	T2DM	Retros.	PBC	ARRAY	WHO	49.7	25.3	0.404	0.459	25.1	21.0
Ng MC (2008) (SNUH)	Asian	T2DM	Retros.	PBC	TaqMan	WHO	59.2	64.7	0.465	0.454	24.5	23.6
Ng MC (2008) (KHGS)	Asian	T2DM	Pros.	PBC	TaqMan	WHO	56.1	55.8	0.536	0.531	25.5	24.2
Omori S (2008)	Asian	T2DM	Retros.	PBC	TaqMan	WHO	61.5	45.5	0.6	0.6	23.7	22.9
Lyssenko V (2008)	Caucasian	T2DM	Pros.	PBC	ARRAY	NA	NA	NA	NA	NA	NA	NA
Sanghera DK (2008)	Asian	T2DM	Retros.	PBC	TaqMan	WHO	54.2	51.3	0.562	0.477	27.8	27.0
Wu Y (2008) a	Asian	T2DM	Retros.	PBC	PCR-related	WHO	59.7	58.4	0.488	0.415	25.1	23.5
Wu Y (2008) b	Asian	T2DM	Retros.	PBC	PCR-related	NA	NA	NA	NA	NA	NA	NA
Wu Y (2008) c	Asian	T2DM	Retros.	PBC	PCR-related	NA	NA	NA	NA	NA	NA	NA
Kirchhoff K (2008) a	Caucasian	T2DM	Retros.	HBC	TaqMan				0.389	0.354		
Kirchhoff K (2008) b	Caucasian	IGT/IFG	Retros.	HBC	TaqMan	NA	NA	NA	0.329	0.354	NA	NA
van Hoek M (2008)	Caucasian	T2DM	Pros.	PBC	TaqMan	NA	73.6	69.0	0.398	0.403	26.8	26.0
van Vliet-Ostaptchouk JV (2008)	Caucasian	T2DM	Retros.	PBC	TaqMan	WHO	70.7	47.8	0.54	0.389	27.60	NA
Cho YM (2009)	Asian	GD	Retros.	PBC	TaqMan	NA	32.0	64.7	1.0	0.45	23.1	23.3
Hu C (2009)	Asian	T2DM	Retros.	PBC	ARRAY	WHO	61.21	57.39	0.525	0.412	24.04	23.57
Lauenborg J (2009)	Caucasian	GD	Pros.	PBC	TaqMan	NA	43.1	45.2	1.0	1.0	28.9	25.0
Pivovarova O (2009) a	Caucasian	T2DM	Retros.	PBC	TaqMan	NA	60.3	53.2	0.464	0.311	31.5	30.0
Pivovarova O (2009) b	Caucasian	IGT/IFG	Retros.	PBC	TaqMan	NA	58.0	53.2	0.264	0.311	29.8	30.0
Rong R (2009)	Asian	T2DM	Retros.	PBC	SNPlex	WHO	37.2	31.1	0.37	0.46	38.5	35.7
Tabara Y (2009)	Asian	T2DM	Retros.	PBC	TaqMan	ADA	60.0	59.0	0.553	0.532	24.0	23.0
Takeuchi F (2009) a	Asian	T2DM	Retros.	PBC	TaqMan	WHO	66.6	64.7	0.607	0.525	24.5	23.3
Takeuchi F (2009) b	Asian	T2DM	Retros.	PBC	TaqMan	WHO	62.7	71.1	0.552	0.535	23.3	23.0
Takeuchi F (2009) c	Asian	T2DM	Retros.	PBC	TaqMan	WHO	62.3	63.8	0.632	0.462	NA	22.6
Chauhan G (2010) a	Asian	T2DM	Retros.	PBC	ARRAY	WHO	53.0	50.0	0.581	0.602	26.7	24.9
Chauhan G (2010) b	Asian	T2DM	Retros.	PBC	ARRAY	WHO	46.0	33.0	0.563	0.529	26.9	19.53
Chidambaram M (2010)	Asian	T2DM	Retros.	PBC	ARRAY	WHO	52.0	38.0	NA	NA	25.0	23.0
Gupta V (2010)	Asian	T2DM	Retros.	HBC	ARRAY	WHO	57.39	53.76	0.665	0.516	28.63	28.56
Han X (2010)	Asian	T2DM	Retros.	PBC	ARRAY	WHO	56.0	58.0	0.527	0.341	25.0	25.0
Lin Y (2010)	Asian	T2DM	Retros.	PBC	ARRAY	WHO	60.2	58.1	0.478	0.5	23.9	23.5
Tan J (2010) a	Asian	T2DM	Retros.	PBC	ARRAY	NA	NA	NA	NA	NA	NA	NA
Tan J (2010) b	Asian	T2DM	Retros.	PBC	ARRAY	NA	NA	NA	NA	NA	NA	NA
Tan J (2010) c	Asian	T2DM	Retros.	PBC	ARRAY	NA	NA	NA	NA	NA	NA	NA
Wen J (2010)	Asian	T2DM	Retros.	PBC	TaqMan	WHO	60.3	59.1	0.391	0.311	25.2	24.1
Xu M (2010) a	Asian	T2DM	Retros.	PBC	SNPshot	WHO	63.3	59.3	0.439	0.384	26.3	24.3
Xu M (2010) b	Asian	IGT/IFG	Retros.	PBC	SNPshot	NA	61.0	59.3	0.4	0.384	25.56	24.3
Zhou DZ (2010)	Asian	T2DM	Retros.	PBC	TaqMan	WHO	63.9	58.1	0.411	0.311	NA	NA
Gutierrez-Vidal R (2011)	Mexican	T2DM	Retros.	HBC	ARRAY	WHO	NA	NA	NA	NA	NA	NA
Kifagi C (2011)	Arabs	T2DM	Retros.	HBC	PCR-related	WHO	64.26	59.78	0.501	0.471	27.07	24.8
Ryoo H (2011)	Asian	T2DM	Pros.	HBC	ARRAY	ADA	56.8	51.9	0.537	0.468	25.2	24.6
Cooke JN (2012)	African	T2DM	Retros.	PBC	ARRAY	NA	59.1	48.75	0.39	0.47	31.7	29.8
Kwak S (2012)	Asian	GD	Retros.	HBC	ARRAY	NA	31.5	59.1	0.0	0.0	23.3	24.6

*Abbreviations:* T2DM, type 2 diabetes mellitus; IGT/IFG, impaired glucose tolerance/impaired fasting glucose; GD, gestational diabetes; Pros., prospective design; Retros., retrospective design; PBC, population-based source of controls; HBC, hospital-based source of controls; WHO, World Health Organization; ADA, American Diabetes Association; BMI, body mass index; NA, not available.

There were 49 studies (43 on type 2 diabetes, 5 on IGT or IFG, 2 on gestational diabetes) encompassing 57931/74658 cases/controls for rs1111875, 18 studies (15 on type 2 diabetes, 1 on IGT or IFG, 2 on gestational diabetes) encompassing 18227/30366 cases/controls for rs5015480, and 26 studies (22 on type 2 diabetes, 3 on IGF or IFG, 1 on gestational diabetes) encompassing 25725/30579 cases/controls for rs7923837. The frequency of rs1111875-C allele in type 2 diabetic patients (45.78%) was lower than that in subjects with IGT/IFG (51.96%) and gestational diabetic patients (48.99%), whereas the frequency of rs5015480-C allele in type 2 diabetic patients (33.53%) was significantly higher than that in subjects with IGT/IFG (17.69%) and gestational diabetic patients (21.85%). In contrast, frequency of rs7923837-G allele in type 2 diabetic patients (41.61%) was intermediate between that of subjects with IGT/IFG (52.66%) and gestational diabetic patients (24.94%). Across ethnicities in type 2 diabetic patients, frequency of rs1111875-C allele in Asians (34.52%) was exceedingly lower than that in Caucasians (61.97%), Mexicans (62.75%), Arabs (74.92%) and Africans (77.6%). Similar tendency was noted for the frequencies of rs5015480-C and rs7923837-G alleles in Asians (26.49% and 25.1%), Caucasians (61.52% and 65.91%) and Africans (92.87%), respectively. There was no detectable deviation from the Hardy-Weinberg equilibrium in all studies but one by van Hoek M et al [48] for rs1111875.

### Overall association

**Figure 2:**
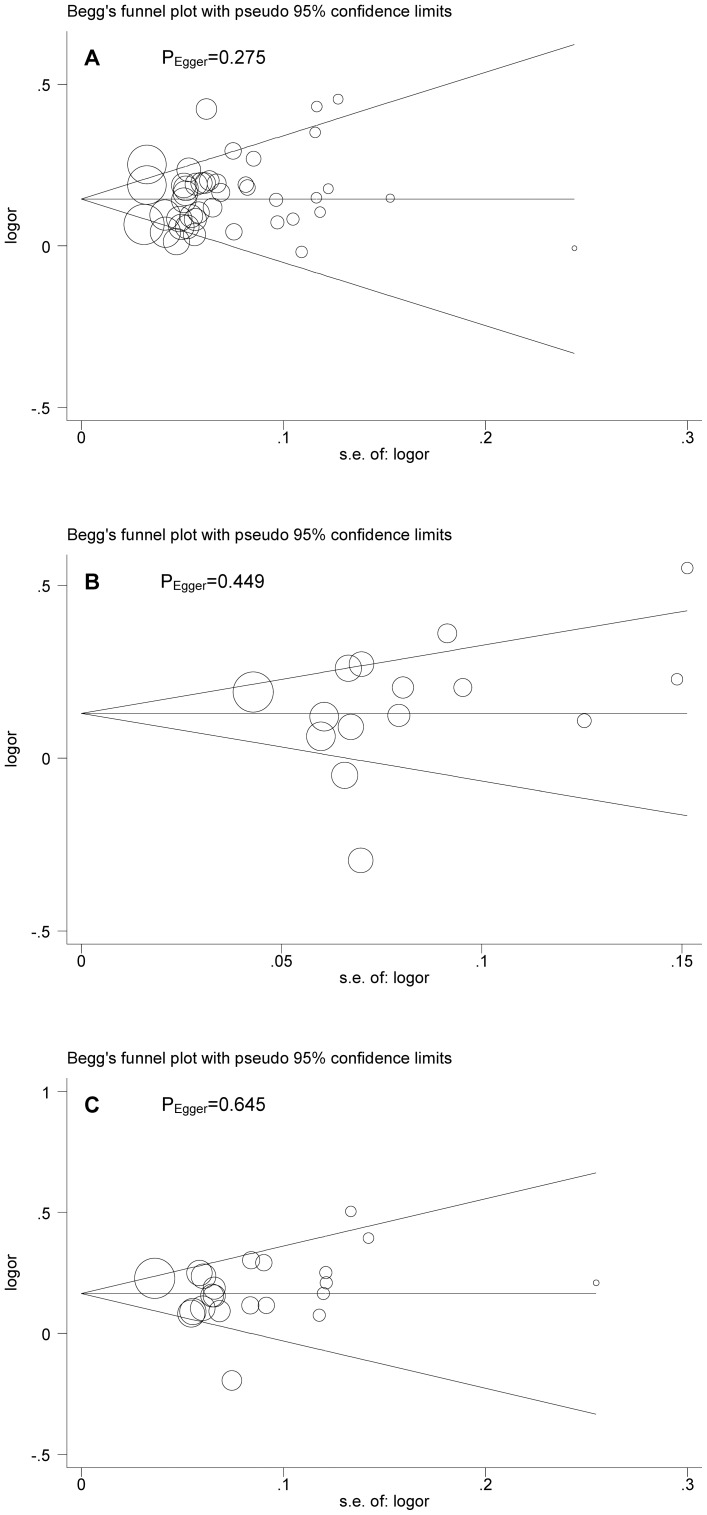
Begg's funnel plot of rs1111875 (A), rs5015480 (B) and rs7923837 (C) polymorphisms in the HHEX gene and type 2 diabetes.

In the main analyses, a random-effects model that takes into account both intra- and inter-study variability indicated that the presence of rs1111875-C (OR = 1.16; 95% CI: 1.13–1.2; P<0.0005), rs5015480-C (OR = 1.16; 95% CI: 1.08–1.25; P<0.0005) and rs7923837-G (OR = 1.18; 95% CI: 1.13–1.24; P<0.0005) alleles was significantly associated with an increased risk of developing diabetes (Figure S1). Restricting studies to type 2 diabetes yielded the very comparable risk estimates for all examined polymorphisms (Table 2). Excluding the study [48] not in Hardy-Weinberg equilibrium yielded exactly the same results. As expected, risk estimates for IGT/IFG was remarkably attenuated and remained non-significant for rs1111875 and rs501548. However, the presence of risk alleles of three examined polymorphisms magnified their association with gestational diabetes, although the involved sample size was relatively small. Beyond overall significant association, there was moderate to strong evidence of between-study heterogeneity. As reflected by the visual Begg's funnel plot and the corresponding Egger's test, there was low probability of publication bias for rs1111875 (P_Egger_ = 0.275), rs5015480 (P_Egger_ = 0.449) and rs7923837 (P_Egger_ = 0.645) (Figure 2).

**Table 2:** Overall and stratified risk estimates of HHEX gene three examined polymorphisms for diabetes mellitus under allelic model.

Overall & subgroups		rs1111875 (C versus T)		rs5015480 (C versus T)		rs7923837 (G versus A)	
		Studies (cases/controls), n(n/n)	OR; 95% CI; P; *I* ^2^ (%)	Studies (cases/controls), n(n/n)	OR; 95% CI; P; *I* ^2^ (%)	Studies (cases/controls), n(n/n)	OR; 95% CI; P; *I* ^2^ (%)
***Total studies***		49 (57931/74658)	1.16; 1.13–1.2; <0.0005; 57.4	18 (18227/30366)	1.16; 1.08–1.25; <0.0005; 77.0	26 (25725/30579)	1.18; 1.13–1.24; <0.0005; 55.6
**T2DM**		43 (54794/68026)	1.16; 1.13–1.2; <0.0005; 59.3	15 (15410/26292)	1.16; 1.06–1.26; 0.001; 80.7	22 (22997/26423)	1.18; 1.12–1.24; <0.0005; 59.7
**IGT/IFG**		5 (1996/3674)	1.09; 0.9–1.32; 0.374; 62.4	1 (1487/2200)	1.1; 0.97–1.24; 0.151; NA	3 (1868/3527)	1.18; 1.02–1.36; 0.024; 39.8
**GDM**		2 (1141/2959)	1.22; 1.08–1.37; 0.001; 0.0	2 (1330/1874)	1.22; 1.07–1.38; 0.003; 0.0	1 (860/629)	1.25; 1.05–1.49; 0.012; NA
***T2DM-Ethnicity***							
Asian	27 (31628/35513)		1.18; 1.15–1.21; <0.0005; 23.2	12 (12156/20949)	1.18; 1.06–1.32; 0.003; 82.5	14 (15484/15733)	1.19; 1.11–1.29; <0.0005; 72.4
Caucasian	12 (18795/29429)		1.14; 1.07–1.22; <0.0005; 79.6	2 (2321/4289)	1.19; 1.11–1.28; <0.0005; 0.0	6 (5750/9132)	1.2; 1.14–1.26; <0.0005; 0.0
African	2 (3585/247)		1.04; 0.95–1.13; 0.401; 0.0	1 (933/1054)	0.95; 0.84–1.8; 0.446; NA	1 (933/1054)	1.18; 0.93–1.49; 0.167; NA
Arabs	1 (331/403)		1.54; 1.23–1.93; <0.0005; NA	NA	NA	1 (830/504)	1.12; 0.94–1.34; 0.209; NA
Mexican	1 (455/234)		1.16; 0.93–1.46; 0.199; NA	NA	NA	NA	NA
***T2DM-Study design***							
Prospective	5 (7861/13754)		1.16; 1.02–1.32; 0.028; 88.5	4 (4564/13574)	1.08; 0.85–1.36; 0.539; 93.0	2 (2320/3294)	1.17; 1.07–1.28; 0.001; 0.0
Retrospective	43 (54794/68026)		1.17; 1.13–1.2; <0.0005; 44.4	11 (10846/12718)	1.19; 1.1–1.29; <0.0005; 65.0	20 (20677/23129)	1.19; 1.12–1.25; <0.0005; 63.5
*T2DM-Source of control*							
Population	36 (50657/63949)		1.15; 1.12–1.19; <0.0005; 60.3	13 (14581/17892)	1.19; 1.11–1.27; <0.0005; 62.6	19 (20712/23649)	1.16; 1.11–1.23; <0.0005; 58.8
Hospital	7 (4136/4077)		1.29; 1.2–1.38; <0.0005; 0.0	2 (829/8400)	0.95; 0.57–1.59; 0.845; 90.2	3 (2285/2774)	1.37; 1.14–1.65; 0.001; 41.7
***T2DM-Genotyping***							
Array	18 (25343/33171)		1.14; 1.1–1.18; <0.0005; 47.8	8 (8099/17691)	1.09; 0.96–1.24; 0.185; 86.1	6 (8986/9447)	1.14; 1.01–1.28; 0.04; 83.1
TaqMan	20 (25497/28596)		1.2; 1.14–1.25; <0.0005; 61.2	4 (5057/4493)	1.26; 1.13–1.4; <0.0005; 47.5	13 (11762/12868)	1.2; 1.14–1.27; <0.0005; 27.5
PCR-related	3 (755/2311)		1.33; 0.97–1.82; 0.073; 81.8	2 (429/1908)	1.38; 0.89–2.13; 0.147; 80.3	2 (424/1908)	1.25; 0.92–1.71; 0.16; 66.8
SNAPShot	1 (1825/2200)		1.06; 0.96–1.17; 0.233; NA	1 (1825/2200)	1.07; 0.95–1.2; 0.294; NA	1 (1825/2200)	1.09; 0.98–1.21; 0.13; NA
SNPlex	1 (1374/1748)		1.06; 0.96–1.17; 0.256; NA	NA	NA	NA	NA
***T2DM-Diagnostic criterion***							
WHO	24 (32529/35997)		1.18; 1.13–1.22; <0.0005; 54.7	8 (10257/9959)	1.18; 1.11–1.26; <0.0005; 27.9	12 (14210/15293)	1.15; 1.07–1.23; <0.0005; 70.5
ADA	2 (4440/4548)		1.22; 1.04–1.42; 0.013; 58.0	1 (613/8221)	0.74; 0.65–0.85; <0.0005; NA	3 (4471/4409)	1.24; 1.16–1.32; <0.0005; 0.0
NA	17 (17825/27481)		1.14; 1.08–1.19; <0.0005; 56.8	6 (4540/8112)	1.21; 1.05–1.39; 0.007; 77.9	7 (4316/6721)	1.26; 1.17–1.36; <0.0005; 0.0
***T2DM-Sample size in cases***							
≥500 subjects	32 (51491/61967)		1.16; 1.12–1.19; <0.0005; 63.4	11 (14332/22767)	1.13; 1.03–1.25; 0.014; 84.4	15 (20930/21554)	1.16; 1.1–1.23; <0.0005; 66.0
<500 subjects	11 (3303/6059)		1.22; 1.11–1.33; <0.0005; 39.8	4 (1078/3525)	1.26; 1.05–1.51; 0.013; 56.2	7 (2067/4869)	1.27; 1.13–1.42; <0.0005; 34.9

*Abbreviations:* T2DM, type 2 diabetes mellitus; IGT/IFG, Impaired glucose tolerance/impaired fasting glucose; GDM, gestational diabetes mellitus; WHO, world health organization; ADA, American diabetes association; NA, not available; OR, odds ratio; 95% CI, 95% confidence interval.

### Subgroup analyses

Considering the fact that few studies relied on IGT/IFG and gestational diabetes and therefrom to eliminate their interference, we merely centered on type 2 diabetes in following analyses. To explore the potential sources of heterogeneity, a set of subgroup analyses were undertaken according to descent of study populations (mainly Asian and Caucasian), study design (prospective and retrospective), source of controls (hospital and population), genotyping method (mainly TaqMan and Array), diagnostic criterion (mainly WHO) and sample size in cases (≥500 subjects and <500 subjects), respectively (Table 2).

Grouping studies by ethnicity showed that there was slight difference for three examined polymorphisms between Asians and Caucasians in association with type 2 diabetes, despite the vast divergence in allele frequencies. For instance, the odds associated with rs1111875-C allele was 1.18 (95% CI: 1.15–1.21; P<0.0005) in Asians and 1.14 (95% CI: 1.07–1.22; P<0.0005) in Caucasians. Likewise, studies in either prospective or retrospective design exhibited comparative association for rs1111875 and rs7923837, and the risk estimate was dramatically reduced to non-significant for rs5015480 in prospective studies (OR = 1.08; 95% CI: 0.85–1.36; P = 0.539).

Upon stratification by source of controls, association was greatly potentiated in studies recruiting controls from hospitals relative to those from populations for rs1111875 and rs7923837, whereas was reduced for rs5015480. Take rs7923837 for example, contrast of G allele versus A allele more than doubled the OR, from 1.37 (95% CI: 1.14–1.65; P = 0.001) in studies with hospital-based controls to 1.16 (95% CI: 1.11–1.23; P<0.0005) in studies with population-based controls. With regard to genotyping methods, studies adopting Array technique had relatively weak association compared with those adopting TaqMan technique for three examined polymorphisms. Concerning the diagnostic criteria for type 2 diabetes, close half of studies did not offer such details, and nearly all remaining studies abided by the WHO criteria with the risk estimates in parallel with that of overall estimates.

When the analyses were restricted to the large studies (≥500 case-patients), summary risk effect was identical to the overall result, whereas analyses of small studies (<500 case-patients) detected an overestimation of the true association, significantly yielding an OR of 1.22 (95% CI: 1.11–1.33; P<0.0005) for rs1111875, 1.26 (95% CI: 1.05–1.51; P = 0.013) for rs5015480 and 1.27 (95% CI: 1.13–1.42; P<0.0005) for rs7923837.

### Cumulative analyses

**Figure 3:**
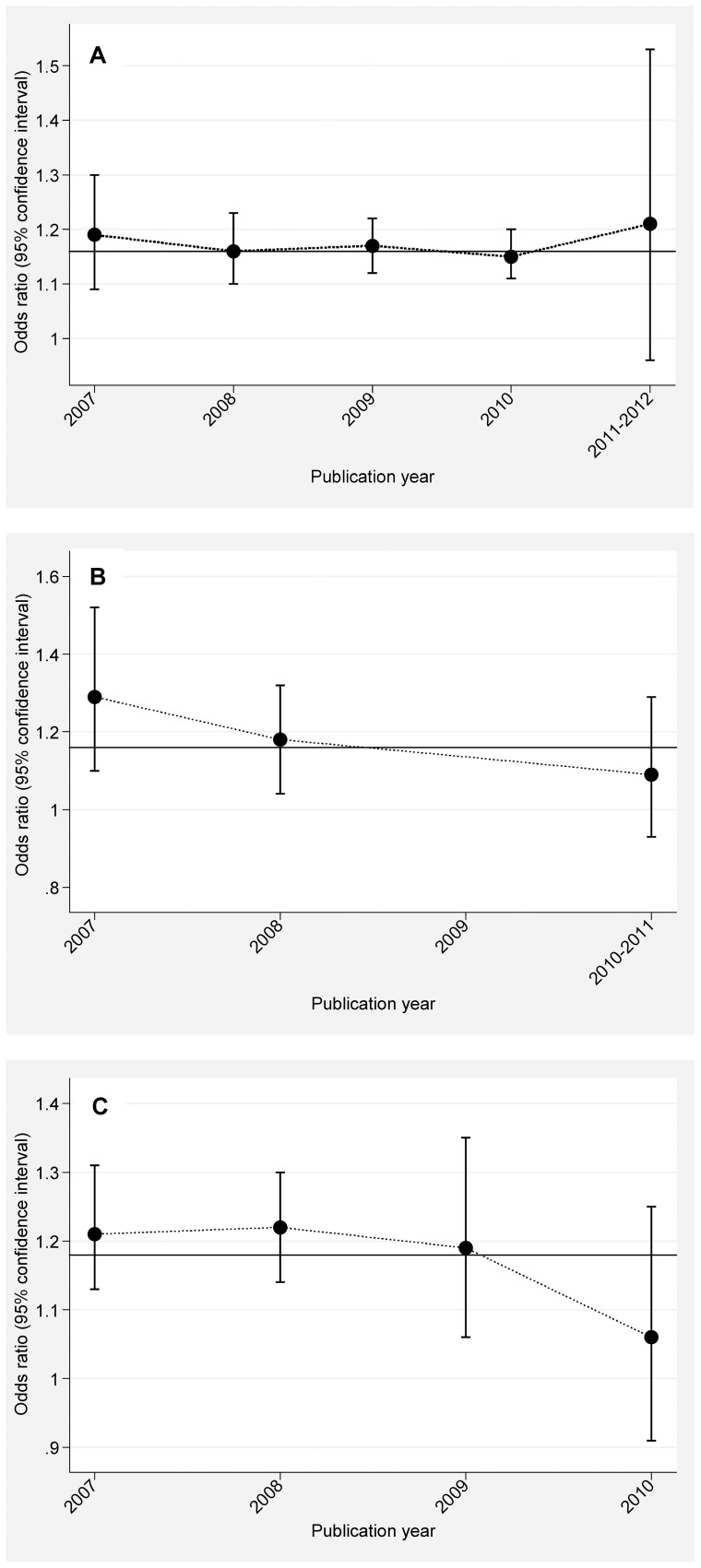
The summary odds ratios examining rs1111875 (A), rs5015480 (B) and rs7923837 (C) in the HHEX gene and type 2 diabetes according to the ascending year of publication.

As shown in Figure 3, summary ORs examining the allelic association of *HHEX* genetic polymorphisms with type 2 diabetes fluctuated around the overall estimates according to the ascending year of publication, and for studies published over the past two years, the risk magnitude tended to be weakened or have wide 95% CIs.

### Meta-regression analysis

In order to further illuminate potential sources of heterogeneity, we undertook a multivariable meta-regression model by incorporating several study-level covariates that were unlikely influenced by drugs, such as age, gender and BMI. However, unfortunately none of these covariates contributed significantly to the association of three examined polymorphisms in *HHEX* gene with type 2 diabetes in a subset of studies that had data these factors.

## Discussion

The present meta-analysis provides the most comprehensive assessment of *HHEX* gene three widely-evaluated polymorphisms and risk of diabetes among 162663 subjects in 43 articles. Importantly, we observed significant association of rs1111875, rs5015480 and rs7923837 in *HHEX* gene with type 2 diabetes. However, the gene effect size suffered from moderate to strong interference of heterogeneity in the results of different studies, reflecting the variation observed between ethnic populations and/or differences in sources of controls. Moreover, when only large studies (≥500 case-patients) were considered, the association remained stable, indicating the robustness of our results.

The pooled results from the Wang et al [49] meta-analysis of rs1111875 and the Cai et al [50] meta-analysis of rs1111875 and rs7923837 in *HHEX* gene with type 2 diabetes were compatible with that of the present meta-analysis. However, given the largest sample sizes, our results provided a relatively precise assessment (ie, narrow CIs) of the average risk estimates. For example, in the Cai et al study [50], the summary per-allele odds ratio for type 2 diabetes of rs7923837 was 1.23 (95% CI: 1.18–1.28) computed from a fixed-effect model, contrasting to the present estimate at 1.18 (95% CI: 1.12–1.24) in random-effects model. Expanding previous findings, we additionally implicated the susceptibility of rs5015480 to type 2 diabetes among 15410 patients and 26292 controls. Besides, our results suggested that the effect estimates associated with three examined polymorphisms were remarkably magnified for gestational diabetes, which required further validation in view of the insufficient samples involved. However, given the consistency of our findings between main analysis and analysis of only the large studies, which are less prone to selective publication and have greater power to detect a true association, we may have full assurance to our observations. Moreover, our risk estimates were also equivalent to that of genome-wide association studies [2], [3].

Another interesting finding of this meta-analysis was that despite the vast differences in allele distributions of examined polymorphisms, subgroup analyses by ethnicity produced comparable results between Asians and Caucasians, which is worthy of our careful thinking. Broadly speaking, distributions of rs1111875-C allele, for example, followed a ladder-like trend of falls from Africans (77.6%) to Caucasians (61.97%) and Mexicans (62.75%), and to Asians (34.52%), in line with a significant degree of gene flow between Africa, Europe and Asia in the famous “Out of Africa” theory. Moreover, the similar effect of rs1111875-C allele on diabetes across ethnicities might be explained by the phenomenon of ‘canalization’, a developmental compensation that can atone for disruptive environmental or genetic forces [51]. Furthermore, considering the complexity of genetic architecture of diabetes, it is assumed that multiple genes take part in the regulation of blood glucose, with each gene exerting a small contribution or interacting with other genes under a certain environmental condition. A polymorphism may be in close linkage with another nearby causal locus in one ethnic population but not in another. It is therefore quite necessary to construct a database of susceptible genes or loci related to diabetes in each race/ethnicity [52].

It is not uncommon to encounter genetic heterogeneity in pooled association studies. Although the overall association of *HHEX* gene with type 2 diabetes reached significance, there was moderate to strong evidence of heterogeneity attributable to genuine changes in gene effect size. In this meta-analysis, source of controls was identified as a potential source of between-study heterogeneity by subgroup analyses for three examined polymorphisms. Notably, the results from studies with hospital-based controls deviated greatly from that of our main analysis. For example, the summary estimate of rs7923837-G allele was at 1.37-fold increased risk for type 2 diabetes in studies with hospital-based controls, a doubling of risk in studies with population-based controls (OR = 1.16) and in all included studies (OR = 1.18). It is widely believed that controls drawn from the general population might be representative of the true population of those without the disease, albeit running the risk of misclassification of study participants. However, studies drawing controls from hospitals had bigger problems in terms of population admixture and stratification, as well as the poor comparability between cases and controls due to their differential hospitalization rates. Another major threat to studies with hospital-based controls was a latent narrow socioeconomic profile, especially drawing controls from only one hospital. Once again, the consistency of our findings between main analysis and analysis of only studies with population-based controls was strong support for the robustness of our observations.

Finally, cautions are urged regarding the interpretation of this meta-analysis. First, most included studies were prospective in design, precluding comments on causality. Second, as with all meta-analyses, publication bias might have occurred because our analyses were based entirely on published studies from English-language journals other than the “grey” literature. Usually, studies with “negative results” either take longer to be published compared with that with enthusiastic results (known as “time lag bias”) or are never published (known as “publication bias”). These biases may have led to an overestimation of the effects in this meta-analysis. Third, although a set of subgroup analyses had been undertaken, significant heterogeneity still persisted in some subgroups, limiting the interpretation of pooled risk estimates. Moreover, considering the relatively small sample sizes for some polymorphisms, especially in subgroups, more studies are warranted to quantify the effect reliably. Fourth, we failed to obtain all of the study-level covariates, preventing a straightforward evaluation of their effects on risk prediction and precluding a more robust assessment of other sources of heterogeneity. Fifth, we only focused on three widely-evaluated polymorphisms in *HHEX* gene, and did not cover other diabetes-susceptibility genes or polymorphisms. It is possible that the potential role of examined polymorphisms is diluted or masked by other gene-gene or gene-environment interactions. Thus, we cannot jump to a conclusion until further verification of our findings in vitro, in vivo and in large prospective studies of multiple gene-gene and gene-environment interactions.

Despites these cautions, our meta-analysis of 162663 subjects provides clarification to the significant association of rs1111875, rs5015480 and rs7923837 in the *HHEX* gene with type 2 diabetes. Moreover, our findings not only confirm and extend the previous meta-analyses, but also indicate obvious robustness in view of stable observations within studies with large sample size and population-based controls. Also our findings leave open the question about the counterintuitive phenomenon for differences strikingly in allele distributions, but negligibly in their risk estimations across ethnicities. Future large, well-designed studies are warranted to provide conclusive evidence on the effects of HHEX gene and other relevant genes on risk of diabetes.

## Supporting Information

Figure S1 **Pooled random-effects odds ratios of developing diabetes for rs1111875 (A), rs5015480 (B) and rs7923837 (C) polymorphisms in the HHEX gene under allelic model.**
(PDF)
