# Supplementary material for: Hematopoietically-Expressed Homeobox Gene Three Widely-Evaluated Polymorphisms and Risk for Diabetes: A Meta-Analysis
Source: PLoS One. 2012 Nov 15;7(11):e49917. doi: 10.1371/journal.pone.0049917 (PMC3499530; doi:10.1371/journal.pone.0049917)
Supplement: Figure S1 — Pooled random-effects odds ratios of developing diabetes for rs1111875 (A), rs5015480 (B) and rs7923837 (C) polymorphisms in the HHEX gene under allelic model. (PDF) [file pone.0049917.s001.pdf]

Supplementary Figure S1. Pooled random-effects odds ratios of developing diabetes for rs1111875 (A), rs5015480 (B) and rs7923837 (C) polymorphisms in the *HHEX* gene under allelic model

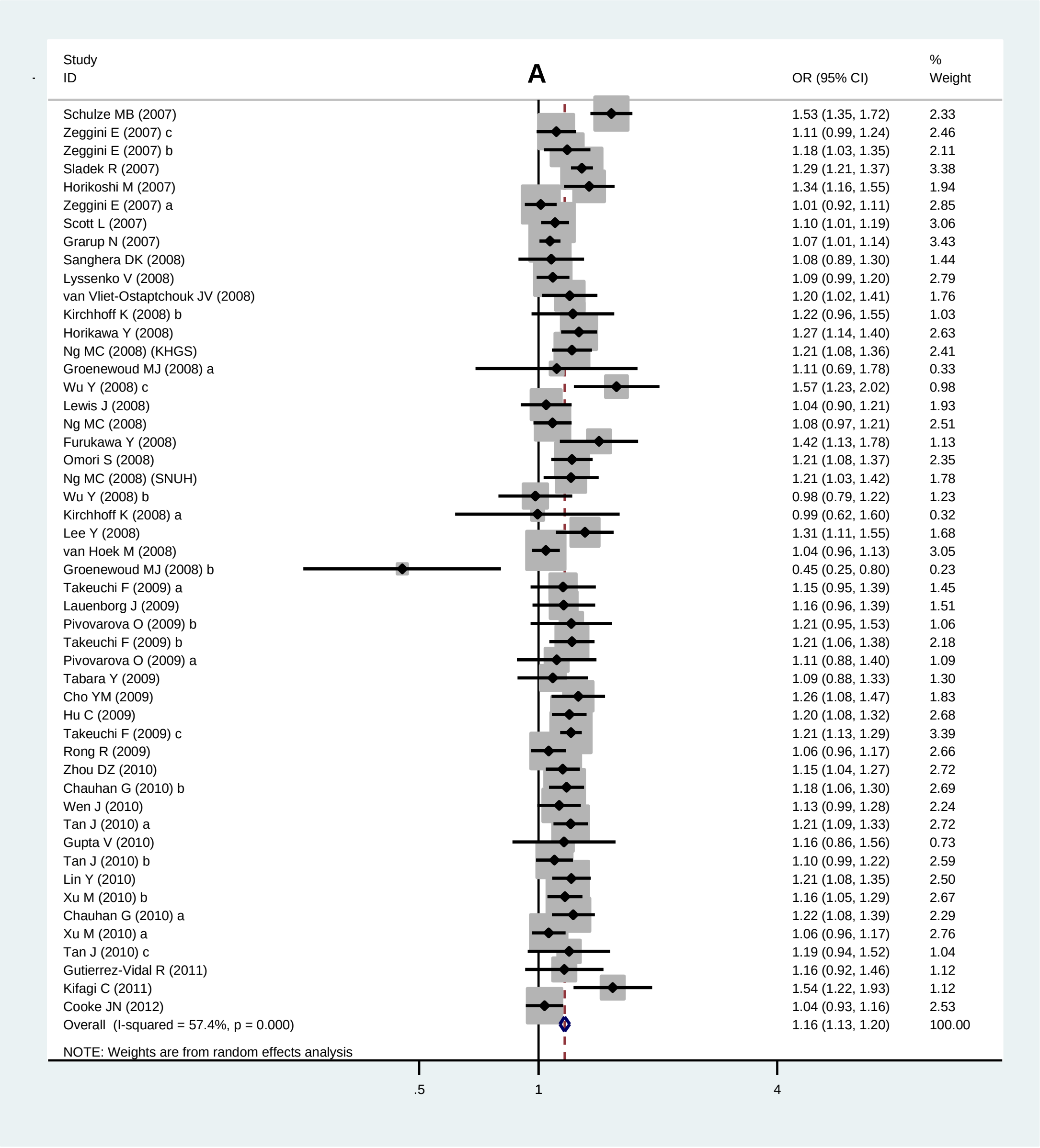

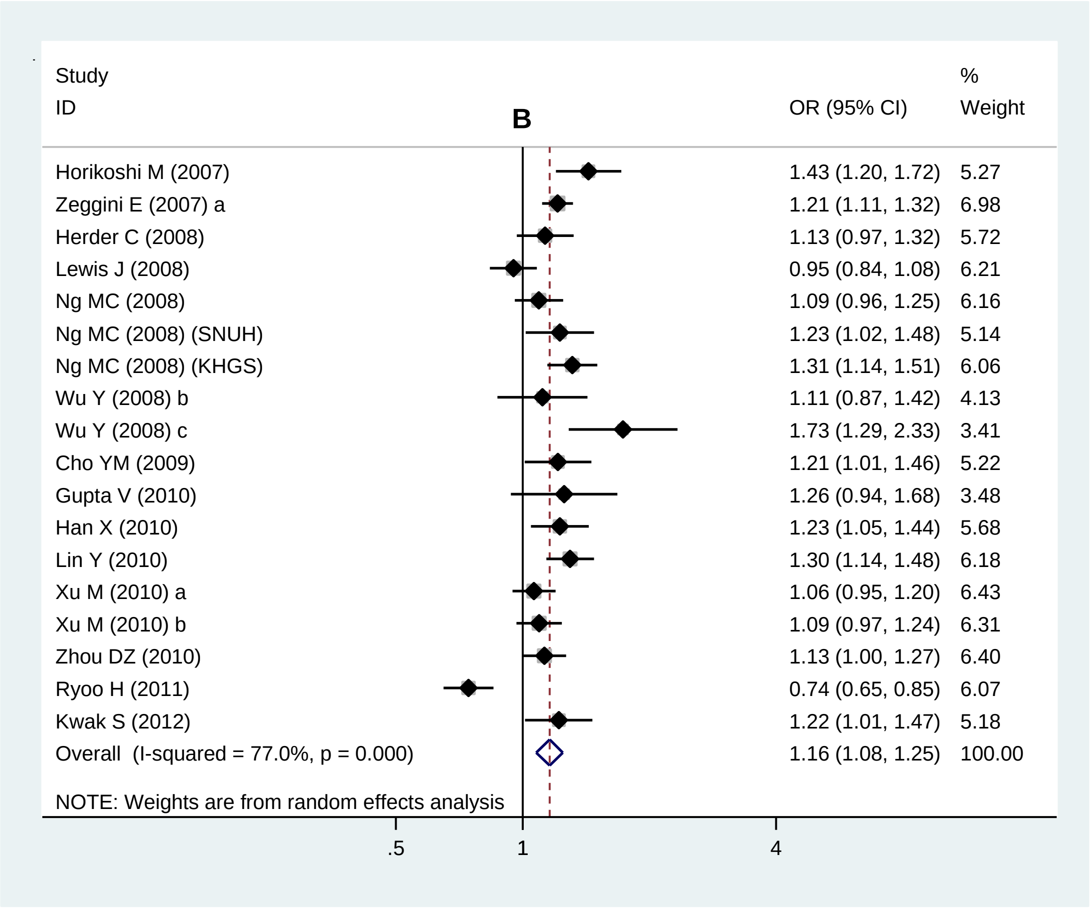
B
Study
ID
OR (95% CI)
%
Weight
Horikoshi M (2007) 1.43 (1.20, 1.72) 5.27
Zeggini E (2007) a 1.21 (1.11, 1.32) 6.98
Herder C (2008) 1.13 (0.97, 1.32) 5.72
Lewis J (2008) 0.95 (0.84, 1.08) 6.21
Ng MC (2008) 1.09 (0.96, 1.25) 6.16
Ng MC (2008) (SNUH) 1.23 (1.02, 1.48) 5.14
Ng MC (2008) (KHGS) 1.31 (1.14, 1.51) 6.06
Wu Y (2008) b 1.11 (0.87, 1.42) 4.13
Wu Y (2008) c 1.73 (1.29, 2.33) 3.41
Cho YM (2009) 1.21 (1.01, 1.46) 5.22
Gupta V (2010) 1.26 (0.94, 1.68) 3.48
Han X (2010) 1.23 (1.05, 1.44) 5.68
Lin Y (2010) 1.30 (1.14, 1.48) 6.18
Xu M (2010) a 1.06 (0.95, 1.20) 6.43
Xu M (2010) b 1.09 (0.97, 1.24) 6.31
Zhou DZ (2010) 1.13 (1.00, 1.27) 6.40
Ryoo H (2011) 0.74 (0.65, 0.85) 6.07
Kwak S (2012) 1.22 (1.01, 1.47) 5.18
Overall (I-squared = 77.0%, p = 0.000) 1.16 (1.08, 1.25) 100.00
NOTE: Weights are from random effects analysis
.5
1
4

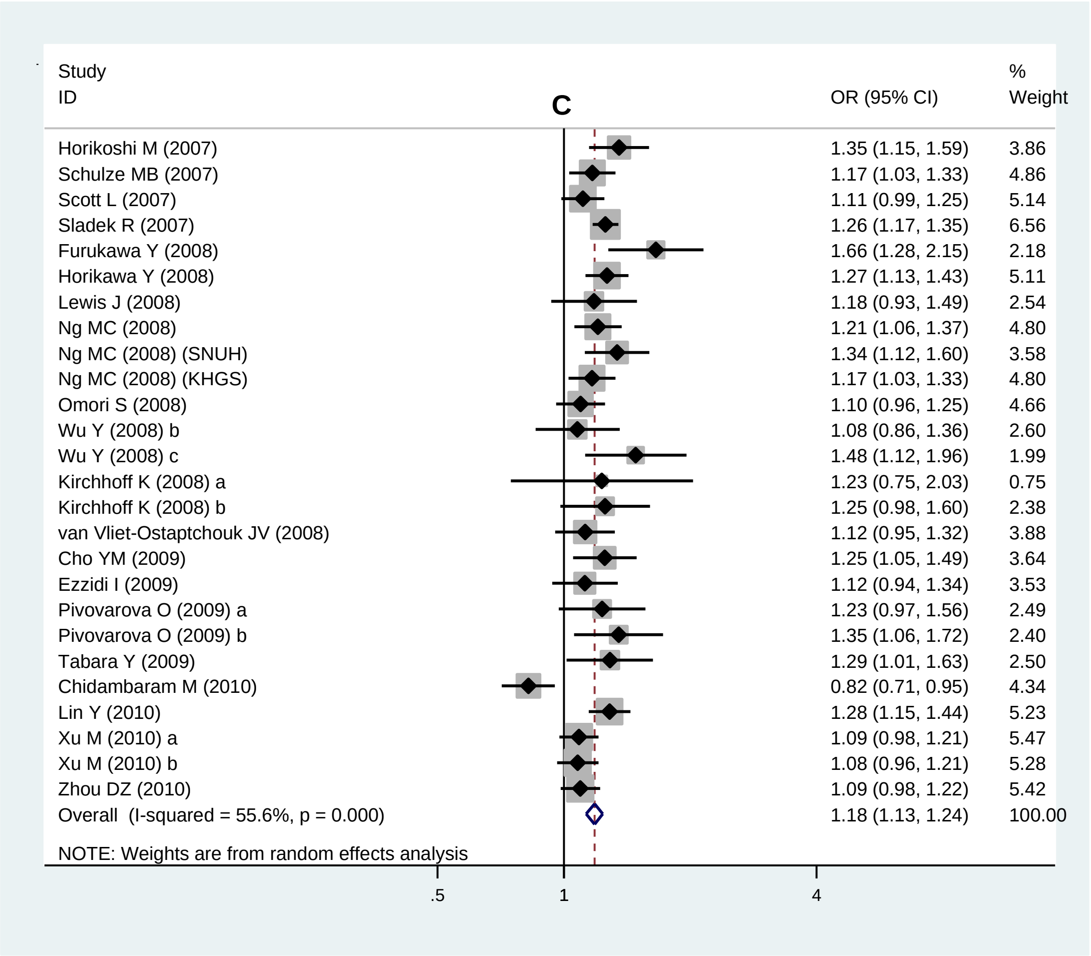
C
Study
ID
OR (95% CI)
%
Weight
Horikoshi M (2007) 1.35 (1.15, 1.59) 3.86
Schulze MB (2007) 1.17 (1.03, 1.33) 4.86
Scott L (2007) 1.11 (0.99, 1.25) 5.14
Sladek R (2007) 1.26 (1.17, 1.35) 6.56
Furukawa Y (2008) 1.66 (1.28, 2.15) 2.18
Horikawa Y (2008) 1.27 (1.13, 1.43) 5.11
Lewis J (2008) 1.18 (0.93, 1.49) 2.54
Ng MC (2008) 1.21 (1.06, 1.37) 4.80
Ng MC (2008) (SNUH) 1.34 (1.12, 1.60) 3.58
Ng MC (2008) (KHGS) 1.17 (1.03, 1.33) 4.80
Omori S (2008) 1.10 (0.96, 1.25) 4.66
Wu Y (2008) b 1.08 (0.86, 1.36) 2.60
Wu Y (2008) c 1.48 (1.12, 1.96) 1.99
Kirchhoff K (2008) a 1.23 (0.75, 2.03) 0.75
Kirchhoff K (2008) b 1.25 (0.98, 1.60) 2.38
van Vliet-Ostaptchouk JV (2008) 1.12 (0.95, 1.32) 3.88
Cho YM (2009) 1.25 (1.05, 1.49) 3.64
Ezzidi I (2009) 1.12 (0.94, 1.34) 3.53
Pivovarova O (2009) a 1.23 (0.97, 1.56) 2.49
Pivovarova O (2009) b 1.35 (1.06, 1.72) 2.40
Tabara Y (2009) 1.29 (1.01, 1.63) 2.50
Chidambaram M (2010) 0.82 (0.71, 0.95) 4.34
Lin Y (2010) 1.28 (1.15, 1.44) 5.23
Xu M (2010) a 1.09 (0.98, 1.21) 5.47
Xu M (2010) b 1.08 (0.96, 1.21) 5.28
Zhou DZ (2010) 1.09 (0.98, 1.22) 5.42
Overall (I-squared = 55.6%, p = 0.000) 1.18 (1.13, 1.24) 100.00
NOTE: Weights are from random effects analysis
.5
1
4
